# Heregulin-expressing HER2-positive breast and gastric cancer exhibited heterogeneous susceptibility to the anti-HER2 agents lapatinib, trastuzumab and T-DM1

**DOI:** 10.18632/oncotarget.12743

**Published:** 2016-10-19

**Authors:** Yoshikane Nonagase, Kimio Yonesaka, Hisato Kawakami, Satomi Watanabe, Koji Haratani, Takayuki Takahama, Naoki Takegawa, Hiroto Ueda, Junko Tanizaki, Hidetoshi Hayashi, Takeshi Yoshida, Masayuki Takeda, Yasutaka Chiba, Takao Tamura, Kazuhiko Nakagawa, Junji Tsurutani

**Affiliations:** ^1^ Department of Medical Oncology, Kindai University Faculty of Medicine, Osakasayama, Osaka, Japan; ^2^ Clinical Research Center, Kindai University Faculty of Medicine, Osakasayama, Osaka, Japan

**Keywords:** HER2, T-DM1, resistance, heregulin, breast cancer

## Abstract

**Background:**

Overexpression of heregulin, a HER3 ligand, is one mechanism that confers resistance to the anti-HER2 agents trastuzumab and lapatinib. We investigated the impact of heregulin expression on the efficacy of HER2-targeted therapeutic agents, including trastuzumab, trastuzumab emtansine (T-DM1) and lapatinib, in vitro and in vivo and evaluated the heregulin messenger RNA (mRNA) levels in specimens from patients with HER2-positive breast or gastric cancer.

**Results:**

Cell proliferation and apoptosis assays demonstrated that heregulin conferred robust resistance to lapatinib and trastuzumab via HER3-Akt pathway activation followed by survivin overexpression; however, heregulin conferred minimal or no resistance to T-DM1 and paclitaxel. The heregulin mRNA level of one of 10 patients was up-regulated after the acquisition of resistance to trastuzumab-based therapy.

**Materials and Methods:**

SK-BR-3, NCI-N87, BT-474, MDA-MB-453, HCC1954, SNU-216 and 4-1ST cells were pharmacologically treated with recombinant heregulin or transfected with the heregulin gene. We also assessed the expression of heregulin mRNA in HER2-positive breast or gastric cancer samples before and after trastuzumab-based therapy using a RT-PCR-based method.

**Conclusions:**

mRNA up-regulation of heregulin was observed in clinical breast cancer specimens during trastuzumab-based treatment, but heregulin overexpression had a limited effect on the sensitivity to T-DM1 in vitro and in vivo.

## INTRODUCTION

The human epidermal growth factor receptor (HER) family consists of receptor-type tyrosine kinases that regulate various cell functions, including cell proliferation, apoptosis, migration and differentiation. HERs are aberrantly activated due to overexpression or activating mutations in some cancer cells [1]. Furthermore, ligand binding to HERs causes a conformational alteration, dimerization and, ultimately, auto- or trans-activation of these receptors [2]. Uniquely among HER family members, HER2 can activate itself via homodimerization without ligand binding, especially in HER2 gene-amplified tumors [3]. In breast and gastric cancer, the HER2 gene is amplified in approximately 20% of patients, and its amplification is closely correlated with the efficacy of anti-HER2 agents [4, 5].

The anti-HER2 agents currently prescribed in clinical settings include lapatinib, trastuzumab and trastuzumab emtansine (T-DM1). These drugs inhibit cancer cell proliferation via a specific and unique mechanism. Lapatinib is a small molecular agent that inhibits the intracellular kinase activity of both EGFR and HER2 [6]. Trastuzumab is a monoclonal antibody that binds to the extracellular domain of HER2 and promotes HER2 internalization [7]. T-DM1, which is composed of a trastuzumab molecule conjugated to DM1—an antimitotic agent that inhibits microtubule polymerization—has characteristics of each component [7, 8].

Heregulin (also known as neuregulin 1 or NRG1) is a ligand for HER3 and HER4 and preferentially induces HER2-HER3 heterodimerization [9, 10]. In clinical settings, HER3 or heregulin overexpression in breast cancer specimens has been correlated with poor prognosis [11, 12]. The heregulin-dependent HER2-HER3 heterodimer was reported to be the most oncogenic HER dimer [13], and this dimer induced resistance of colorectal cancer to anti-EGFR antibodies and of non-small cell lung cancer to EGFR tyrosine kinase inhibitors [14, 15]. However, in HER2-positive breast and gastric cancer, the effects of heregulin on various anti-HER2 agents, especially T-DM1, have not been fully elucidated.

The aim of the current study was to assess the effect of heregulin on the efficacy of the anti-HER2 agents trastuzumab, lapatinib and especially T-DM1 in breast and gastric cancer. We conducted in vitro experiments utilizing HER2-positive breast and gastric cell lines pharmacologically treated with recombinant heregulin or transfected with the heregulin gene. We also assessed the expression of heregulin mRNA in clinical specimens obtained from patients with HER2-positive breast or gastric cancer before and after trastuzumab-based therapy.

## RESULTS

### Heregulin promoted Akt phosphorylation in the SK-BR-3 and NCI-N87 cell lines

**Figure 1:**
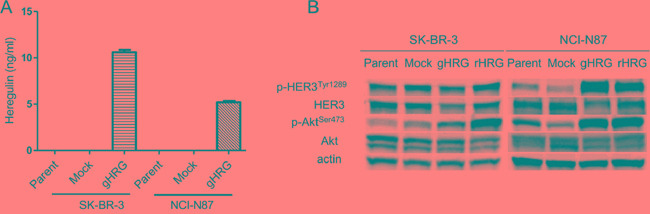
Characterization of cell lines **A.** SK-BR-3 and NCI-N87 cell lines and their derivatives (Mock, gHRG) were seeded in 12-well plates at a density of 0.5×10^6^ cells per well in RPMI medium supplemented with 10% FBS overnight. These cells were then incubated for 48 hours in RPMI medium supplemented with 0.1% FBS, and the heregulin levels in the culture supernatants were assessed via ELISA. **B.** SK-BR-3 and NCI-N87 cell lines and their derivatives (Mock and gHRG) were cultured overnight in medium containing 10% FBS and then incubated for 48 hours in medium containing 2% FBS. Recombinant heregulin was added to parental SK-BR-3 and NCI-N87 cells at 20 ng/ml (rHRG). After 15 minutes, the cells were lysed and subjected to immunoblotting analysis. Data are presented as the mean ± SE of three independent experiments. Mock=empty vector-transfected cells, gHRG=*heregulin*-transfected cells, rHRG=recombinant heregulin-treated parental cells.

To confirm that SK-BR-3 and NCI-N87 cells transfected with the heregulin gene expressed heregulin protein, we conducted an immunoblot assay utilizing cell lysates of SK-BR-3 or NCI-N87 lines that were genetically engineered to stably overexpress heregulin (SK-BR-3 HRG and NCI-N87 HRG, respectively) or to stably harbor the corresponding empty vector (SK-BR-3 Mock and NCI-N87 Mock, respectively). Heregulin is synthesized as a transmembrane precursor molecule of 105 kDa. The extracellular domain of heregulin (44 kDa) is cleaved by cell surface proteases and functions as a ligand for HER3, leaving a transmembrane region of approximately 60 kDa. An immunoblot assay revealed the presence of the 2 transmembrane forms of heregulin in the HRG cell lines (105 kDa and 60 kDa), but these bands were not detected in the Mock or parental cell lines (supplemental Figure 1). Furthermore, analysis of cell culture supernatants via enzyme-linked immunosorbent assays (ELISAs) revealed heregulin at concentrations of 10.6 ng/ml in SK-BR3 HRG cells and 5.2 ng/ml in NCI-N87 HRG cells; in contrast, heregulin was undetectable in the other cell lines (Figure 1A). Furthermore, an immunoblot assay was conducted to determine how heregulin affects downstream signaling. In cell lines transfected with heregulin or exposed to recombinant heregulin, Akt phosphorylation (Ser473) was elevated compared with that of cell lines not transfected with heregulin or treated with recombinant heregulin. HER3 phosphorylation (Tyr1289) was elevated in heregulin-exposed NCI-N87 cells but not in SK-BR-3 cells (Figure 1B).

### Heregulin conferred robust resistance to lapatinib and trastuzumab but not T-DM1 or paclitaxel

**Figure 2:**
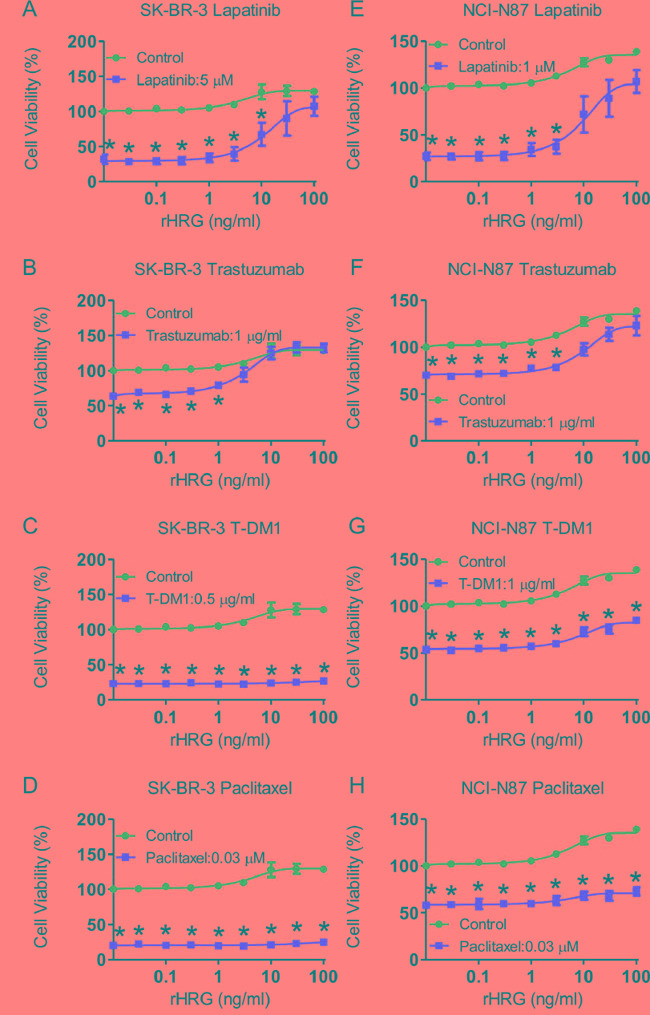
Addition of recombinant heregulin and drug resistance in SK-BR-3 and NCI-N87 cell lines SK-BR-3 cells **A-D.** and NCI-N87 cells **E-H.** were incubated for 72 hours in the presence of anticancer drugs (red line) or absence of those drugs (blue line) with increasing doses of recombinant heregulin, and cell viability was measured. The fixed doses of lapatinib (5 μM for SK-BR-3, 1 μM for NCI-N87), trastuzumab (1 μg/ml), T-DM1 (0.5 μg/ml for SK-BR-3, 1 μg/ml for NCI-N87) and paclitaxel (0.03 μM) were the lowest dose that resulted in maximum growth inhibition. The p values were calculated using an unpaired Student's t-test, where * indicates a p value < 0.0055 as determined by the Bonferroni correction for multiple comparisons. rHRG=recombinant heregulin.

To determine whether heregulin affects drug susceptibility in HER2-positive cell lines, we added increasing concentrations of recombinant heregulin to the medium of SK-BR-3, NCI-N87, BT-474, MDA-MB-453, HCC1954, SNU216 and 4-1ST cells in the presence or absence of lapatinib, trastuzumab, T-DM1 and paclitaxel. As the recombinant heregulin concentration increased to 100 ng/ml, cell viabilities increased to varying degrees, depending on the types of anticancer agent and the cell line (Figure 2, supplemental Figure 2). Although the viability of cells treated with lapatinib and trastuzumab significantly increased to levels equivalent to those of the vehicle-treated cells as the recombinant heregulin concentration increased (Figure 2A, 2B, 2E, 2F, supplemental Figure 2A, 2B, 2E, 2F, 2N, 2R), the viability of cells treated with T-DM1 or paclitaxel did not show similar increases (Figure 2C, 2D, 2G, 2H, supplemental Figure 2D, 2G, 2H, 2K, 2L, 2P, 2S, 2T). Interestingly, heregulin did not desensitize HCC1954, SNU-216 and 4-1ST cells to lapatinib (supplemental Figure 2I, 2M, 2Q), and HCC1954 cells were resistant to trastuzumab regardless of the presence of heregulin (supplemental Figure 2J). BT-474 and SNU-216 cells treated with T-DM1 showed significantly increased cell viability following recombinant heregulin treatment, although the extent was less than that with lapatinib or trastuzumab (supplemental Figure 2A, 2B, 2C, 2N, 2O). These results indicated that although recombinant heregulin conferred robust resistance to lapatinib and trastuzumab, it promoted T-DM1 resistance to a much lesser extent in HER2-positive cancer cells.

**Figure 3:**
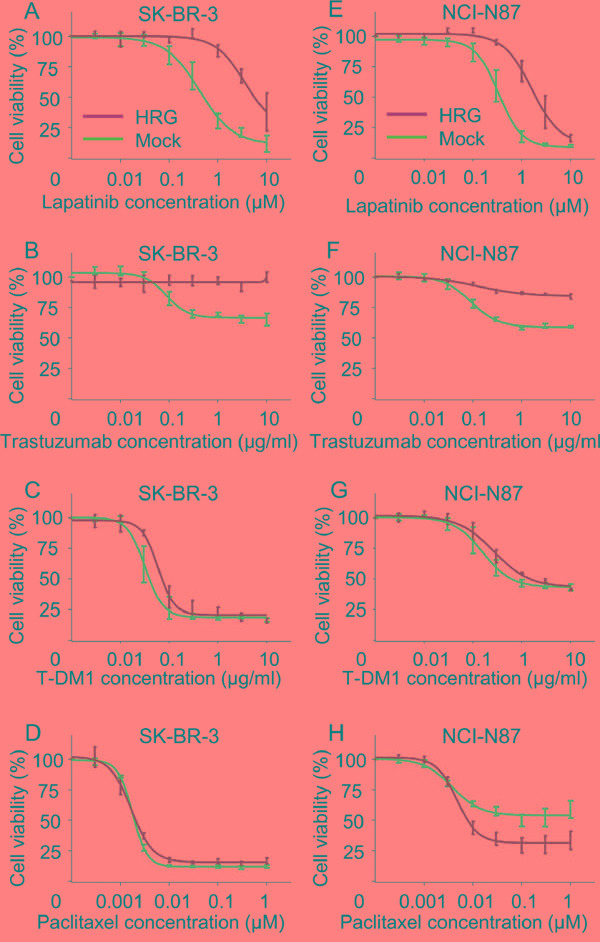
In vitro growth inhibition assay in heregulin-transfected cells in the presence of lapatinib, trastuzumab, T-DM1 or paclitaxel The inhibitory effects of lapatinib, trastuzumab, T-DM1 and paclitaxel were evaluated in SK-BR-3 **A-D.** and NCI-N87 **E-H.** Mock (blue lines) and HRG cells (green lines). The cells were incubated for 72 hours with the indicated drugs, and cell viability was measured. Data are presented as the mean ± SE of three independent experiments.

To confirm the observed effects of exogenous heregulin on drug susceptibility, we also conducted growth inhibition assays using the SK-BR-3 and NCI-N87 Mock and HRG cell lines (Figure 3). HRG cells were less sensitive to lapatinib and trastuzumab than Mock cells (Figure 3A, 3B, 3E, 3F). Susceptibility to T-DM1 was also reduced; however, the extent of this reduction was much less than that for lapatinib or trastuzumab (Figure 3C, 3G). The susceptibility of SK-BR-3 HRG cells to paclitaxel was similar to that of SK-BR-3 Mock cells (Figure 3D), and the susceptibility of NCI-N87 HRG cells was greater than that of NCI-N87 Mock cells (Figure 3H).

### Elevated phosphorylation of Akt in SK-BR-3 HRG cells led to resistance to lapatinib and trastuzumab but not T-DM1 or paclitaxel

**Figure 4:**
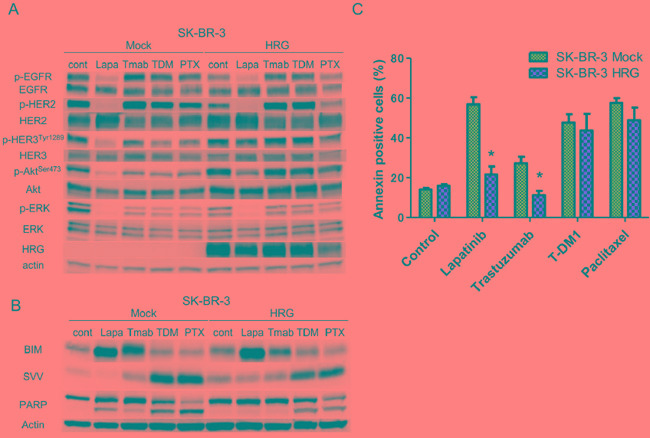
Effects of anticancer drugs on intracellular signaling and apoptosis in the SK-BR-3 Mock and HRG cell lines SK-BR-3 Mock and HRG cells were cultured overnight in medium containing 2% FBS and then incubated for 24 hours **A.** or 48 hours **B, C.** with conditioned media in the presence of lapatinib (5 μM), trastuzumab (1 μg/ml), T-DM1 (0.5 μg/ml) or paclitaxel (0.03 μM). (A, B). Whole cell lysates were prepared and subjected to immunoblotting analysis using antibodies against the indicated proteins. (C). The number of apoptotic cells was determined by staining with fluorescein isothiocyanate (FITC)-labeled Annexin V followed by flow cytometry. Data are presented as the mean ± SE of three independent experiments. * indicates p value < 0.05 vs Mock, unpaired t-test. Cont=control. Lapa=lapatinib. Tmab=trastuzumab. PTX=paclitaxel. HRG=heregulin. SVV=survivin.

The effect of heregulin on the susceptibility of SK-BR-3 and NCI-N87 cells to T-DM1 was very different from its effects on susceptibility to lapatinib or trastuzumab, although all of these drugs target HER2. Next, we conducted immunoblot assays to investigate possible differences in signal transduction and performed fluorescence-activated cell sorting (FACS) to assess apoptosis in SK-BR3 cell lines. Evaluation of intracellular signaling revealed that phosphorylation of HER3 and Akt was up-regulated in HRG cells compared with that in Mock cells in the presence of all anticancer agents, but phosphorylation of EGFR, HER2 and ERK was similar between Mock and HRG cells (Figure 4A). FACS analysis revealed a significant reduction in Annexin-positive cells in HRG cell lines treated with lapatinib or trastuzumab. In contrast, little change in the percentage of Annexin-positive cells was observed following treatment with T-DM1 or paclitaxel (Figure 4C). Consistent with these results, the levels of the cleaved form of poly (ADP-ribose) polymerase (PARP), which is a marker of apoptosis, were drastically reduced in HRG cells treated with lapatinib or trastuzumab but remained largely unchanged in HRG cells treated with T-DM1 or paclitaxel (Figure 4B). We further evaluated the expression of the apoptosis-related proteins BIM (a proapoptotic BH3-only protein) and survivin (a member of the inhibitor of apoptosis, or IAP, family). Although the BIM levels were similar between Mock and HRG cells, the survivin levels were up-regulated in HRG cells treated with lapatinib or trastuzumab but down-regulated in HRG cells treated with T-DM1 or paclitaxel compared to those of Mock cells (Figure 4B).

### T-DM1 showed efficacy in heregulin-overexpressing NCI-N87 xenografts in vivo

**Figure 5:**
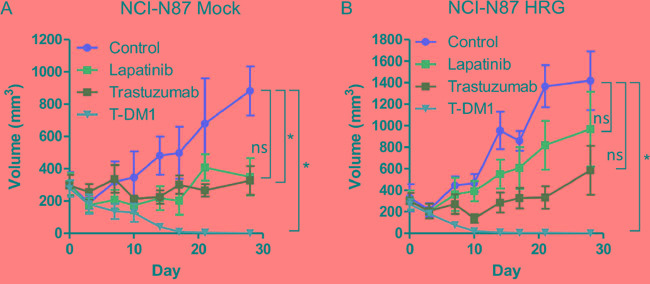
In vivo antitumor efficacy of lapatinib, trastuzumab and T-DM1 in NCI-N87 cells Mice bearing NCI-N87 Mock cell**A.** and HRG cell **B.** xenograft tumors were intraperitoneally administered PBS (100 μl) twice per week and HPMC (100 μl) once daily by oral gavage (control); lapatinib (100 mg/kg) once daily by oral gavage; trastuzumab (10 mg/kg) twice per week intraperitoneally; or T-DM1 (10 mg/kg) as a single intraperitoneal injection. Each treatment group consisted of five mice. Data are presented as the mean ± SE. The p values were calculated using an unpaired Student's t-test, where * indicates p value < 0.0166 as determined by the Bonferroni correction for multiple comparisons. ns=not significant.

To determine whether T-DM1 is effective in a heregulin-overexpressing xenograft model, we injected nude mice with NCI-N87 Mock and HRG cells. Whereas lapatinib or trastuzumab inhibited the growth of NCI-N87 Mock xenografts (Figure 5A), NCI-N87 HRG xenografts were resistant to these drugs (Figure 5B). T-DM1 markedly inhibited the growth of both NCI-N87 Mock and HRG xenografts (Figure 5A, 5B). These results indicated that heregulin-overexpressing tumors were resistant to lapatinib or trastuzumab but sensitive to T-DM1.

### Heregulin mRNA levels were assessed in clinical tissue specimens before and after trastuzumab-based therapy

**Figure 6:**
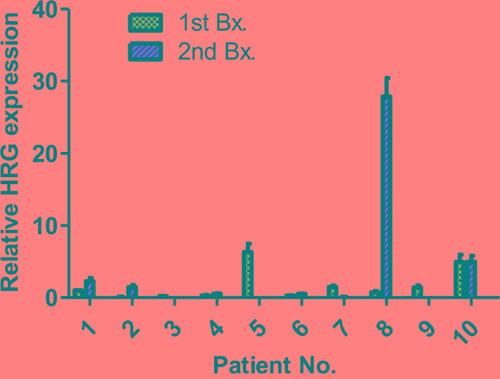
mRNA levels in specimens from patients with breast or gastric cancer mRNA levels were assessed in 8 breast cancer patients and 2 gastric cancer patients. First, biopsy or resection was conducted before trastuzumab-based therapy. Patients no. 1 and no. 2 had a second Bx after trastuzumab-based neoadjuvant chemotherapy and the others after adjuvant and/or palliative trastuzumab-based therapy. The heregulin expression values are normalized to β-actin and relative to the median. Data are presented as the mean ± SE of three independent experiments. The patient ID numbers and abbreviations used correspond to those described in table 1

The mRNA levels of heregulin normalized to the β-actin levels in specimens before and after trastuzumab-based therapy were assessed in 8 breast cancer patients and 2 gastric cancer patients (Table 1, Figure 6). Tissue specimens before trastuzumab-based therapy were obtained in the first biopsy or resection from all patients. The second biopsy or resection was conducted after neoadjuvant trastuzumab-based therapy (patients no. 1 and 2) and after adjuvant and/or palliative trastuzumab-based therapy (patients no. 3-10). Biopsy or surgical resection was performed on tumors in the breast, chest wall or brain among breast cancer patients or in the stomach among gastric cancer patients. T-DM1 was administered only to patient no. 8, pertuzumab was administered to patients no. 7 and no. 8, and lapatinib was not administered to any of the patients before the second biopsy or resection.

**Table 1:** Clinical characteristics of patients with HER2-positive breast or gastric cancer

No.	Age	Sex	Cancer type	Treatment		Bx site	
				Before 1st Bx	Before 2nd Bx	1st	2nd
1	70	F	Breast	-	NAC (Tmab/DTX;PR)	breast	breast
2	49	F	Breast	-	NAC (Tmab/Cape;PR, Tmab/VNR;PR)	breast	breast
3	70	F	Breast	-	NAC (AC;SD) ADJ (Tmab/PTX)	breast	brain
4	46	F	Breast	-	ADJ (Tmab, TMX)	breast	breast
5	66	F	Breast	-	PAL (Tmab/PTX;SD, Tmab/Cape;PR, AC;SD, Tmab/VNR;PD)	breast	brain
6	46	F	Breast	ADJ (CMF, Zoladex/TMX)PAL(TMX/Goserelin;SD)	PAL (Tmab;NA, Tmab/TC;NA, TMX/leuprorelin;PD)	breast	chest wall
7	53	F	Breast	-	PAL (Tmab/PER/PTX;PR)	breast	brain
8	62	F	Breast	-	PAL (Tmab/PTX;PR, Tmab/letrozole;PD, Tmab/PTX;PR, Tmab/Exemestane;SD, TMX;SD, Cape/Tmab;SD, Tmab/PER/DTX;SD, T-DM1;PR)	breast	breast
9	66	M	Gastric	-	ADJ (S-1)PAL (Cape/Tmab;PR)	stomach	brain
10	66	M	Gastric	-	PAL (Cape/CDDP;PR, Tmab/Cape;PD, IRI;PD)	stomach	stomach

Characteristics of patients whose heregulin mRNA levels were assessed both before and after trastuzumab-based therapy. Eight breast cancer patients and 2 gastric cancer patients were evaluated. Tmab=trastuzumab. Pmab=pertuzumab. NAC=neoadjuvant chemotherapy. ADJ=adjuvant chemotherapy. PAL=palliative chemotherapy. Tmab=trastuzumab. PER=pertuzumab. DTX=docetaxel. PTX=paclitaxel. VNR=vinorelbine. Cape=capecitabine. CDDP=cisplatin. TMX=tamoxifen. IRI=irinotecan. CMF=cyclophosphamide/methotrexate/5-FU. TC=docetaxel/cyclophosphamide. AC=doxorubicin/cyclophosphamide. Bx=biopsy or resection. PR=partial response. SD=stable disease. PD=disease progression. NA=not applicable.

Patients no. 1 and 2 received only trastuzumab-based neoadjuvant therapy before the second biopsy, and higher heregulin levels were observed compared with those in the first biopsy specimen. Patient no. 5 displayed relatively high heregulin expression in the first specimen, but the heregulin expression level was decreased in the second biopsy specimen. The second specimen from patient no. 8 displayed the highest heregulin level of all specimens, and this patient was treated with trastuzumab, T-DM1 and pertuzumab before the second biopsy. Patient no. 10 displayed relatively high levels of heregulin both before and after trastuzumab-based therapy.

## DISCUSSION

The current study revealed that recombinant heregulin caused resistance of HER2-positive breast and gastric cancer cells to lapatinib and trastuzumab. Similarly, others have reported that recombinant heregulin treatment induces resistance to these agents [16, 17]. We demonstrated that in addition to the cells treated with exogenous heregulin, the heregulin gene-transfected cells exhibited resistance to these agents in vitro and in vivo, although heregulin-induced resistance to trastuzumab was relatively weak in vivo, which may be due to antibody-dependent cell-mediated cytotoxicity (ADCC) [18]. Therefore, breast or gastric tumors displaying high heregulin levels may be clinically resistant to these agents. In fact, tumor samples obtained from one breast cancer patient after acquisition of resistance to anti-HER2 agents, including trastuzumab, displayed extremely high levels of heregulin mRNA. In addition, specimens obtained from two other patients (no. 5 and no. 10) before trastuzumab-based therapy had relatively high heregulin expression levels, and the clinical response to the initial trastuzumab-based therapy was stable disease in patient no. 5 and disease progression in patient no. 10. Based on these results, heregulin may have clinical relevance in de novo and acquired trastuzumab resistance.

In contrast to lapatinib and trastuzumab, susceptibility to T-DM1 was sustained even in the presence of heregulin. Additionally, reports of heregulin-induced resistance to T-DM1 are limited [19]. A previous clinical trial demonstrated that the anti-HER2 antibody pertuzumab, which inhibits the heterodimerization of HER2 with HER3, was beneficial when combined with trastuzumab plus docetaxel in patients with HER2-positive breast cancer [20]. In contrast, the addition of pertuzumab to T-DM1 did not improve progression-free survival among HER2-positive breast cancer patients in the MARIANNE (TDM4788g/BO22589) trial. Overall, these observations suggested that HER2-HER3 coupling, which depends on heregulin, caused resistance to trastuzumab but not T-DM1. The mechanism underlying heregulin-induced resistance to anti-HER2 agents, including trastuzumab, has been reported to involve HER3-PI3K-Akt pathway activation [16, 17]. Furthermore, the elevated phosphorylation of Akt and subsequent up-regulation of survivin expression were suggested to promote the resistance of breast cancer to lapatinib and of colorectal cancer to cetuximab [14, 21]. In the current study, survivin expression and Akt phosphorylation were elevated in HRG cells treated with lapatinib or trastuzumab compared with those in identically treated Mock cells, although HER3 phosphorylation was not necessarily concomitant with heregulin overexpression, possibly due to a potential negative feedback loop for Akt [22].

To determine why heregulin did not induce substantial resistance to T-DM1, we utilized paclitaxel, which is another antimitotic agent that is often prescribed to breast and gastric cancer patients. In SK-BR-3 Mock and HRG cells treated with T-DM1 or paclitaxel, survivin expression was increased but phosphorylation of Akt was unchanged compared with those of the corresponding control cells. Survivin has a dual role as an apoptosis inhibitor in the cytoplasm and a mitotic effector in the nucleus [23]. Survivin expression as a mitotic effector is regulated by the cell cycle, with an increase in the G2/M phase of the cell cycle. The G2/M arrest caused by paclitaxel and T-DM1 could explain the induction of survivin as a mitotic effector in cells treated with paclitaxel and T-DM1 [24–26]. In HRG cells treated with T-DM1 and paclitaxel, survivin expression was not further increased despite an increase in phosphorylation of Akt compared with that of Mock cells. The unchanged survivin expression may be one reason why heregulin did not induce substantial resistance to T-DM1. However, further study is needed to confirm this mechanism.

A limitation of this study is that the in vitro experiments did not take into consideration extracellular environmental conditions, such as ADCC or the tumor microenvironment. The in vivo experiments used a small number of mice. Furthermore, mechanisms of resistance to trastuzumab in addition to heregulin overexpression, such as PIK3CA mutations or loss of PTEN, were not comprehensively evaluated in the clinical specimens.

In conclusion, susceptibility to T-DM1 depended on HER2 expression but was not affected by heregulin-induced HER2-HER3 heterodimerization followed by downstream signal activation. Heregulin may have clinical relevance in de novo and acquired trastuzumab resistance, but further evaluation of heregulin expression in patients treated with trastuzumab, lapatinib or T-DM1 is necessary to confirm these observations.

## MATERIALS AND METHODS

### Cells and reagents

The SK-BR-3, BT-474 (K111N *PIK3CA* mutant), MDA-MB-453 (H1047R *PIK3CA* mutant, E307K *PTEN* mutant), HCC1954 (H1047R *PIK3CA* mutant) and NCI-N87 cell lines were obtained from the American Type Culture Collection (VA, USA). The SNU-216 cell line was obtained from the Korean Cell Line Bank (Seoul, South Korea) and 4-1ST from the Resource Center for Biomedical Research, Institute of Development, Aging and Cancer Tohoku University (Miyagi, Japan). All cell lines were maintained under a humidified atmosphere of 5% CO_2_ in air at 37°C in RPMI-1640 medium (Sigma-Aldrich, MO, USA) supplemented with 10% fetal bovine serum (FBS). T-DM1 was provided by Chugai Pharmaceuticals Co., Ltd. (Tokyo, Japan). Recombinant human heregulin (NRG1-β1/HRG1-β1 EGF domain) was obtained from R&D Systems (MN, USA). Lapatinib, trastuzumab and paclitaxel were obtained from commercial sources.

### Construction of heregulin-overexpressing cell lines

The full-length cDNA of human heregulin (NRG1, GenBank accession no. NM_013956) was obtained from Origene (MD, USA). The DNA fragment was cloned into the pCR-Blunt II-TOPO vector (Thermo Fisher Scientific, MA, USA), and the presence of amplified heregulin was confirmed by DNA sequencing. The heregulin fragment was transferred to the pQCXIH retroviral vector (Clontech, CA, USA) [14]. Retroviruses encoding heregulin were produced and used to infect SK-BR-3 and NCI-N87 cells [27]. Cells expressing heregulin (SK-BR-3 HRG, NCI-N87 HRG) and cells harboring the corresponding empty vector (SK-BR-3 Mock, NCI-N87 Mock) were isolated via selection with 500 μg/ml hygromycin B (InvivoGen, CA, USA).

### Detection of heregulin expression via immunoblot analysis and ELISA

Cells were cultured in 60-mm plates (Sumitomo Bakelite, Tokyo, Japan) at a density of 1.5×10^6^ cells per plate for 48 hours in RPMI medium supplemented with 2% FBS to assess heregulin expression in the cell lines. Then, the cell lysates were prepared for immunoblot assays [28] using antibodies against phosphorylated Akt, heregulin (both from Cell Signaling Technology, MA, USA) and β-actin (Sigma-Aldrich, MO, USA).

For confirmation of heregulin in cell culture supernatants, cells were seeded in 12-well plates at a density of 0.5×10^6^ cells per well in RPMI medium supplemented with 10% FBS. After incubation overnight, the medium was replaced with 1 ml of RPMI medium supplemented with 0.1% FBS. The cells were incubated for an additional 48 hours, and the culture supernatants were collected. The concentration of heregulin in cell culture supernatants was measured using the Human NRG1 beta 1 ELISA Kit (Abcam, Cambridge, UK) according to the manufacturer's instructions.

### Effect of recombinant heregulin application on SK-BR-3, NCI-N87, BT-474, MDA-MB-453, HCC1954, SNU216 and 4-1ST cells treated with lapatinib, trastuzumab, T-DM1 or paclitaxel

Cells were seeded into 96-well flat-bottom plates at 5.0×10^3^ cells per well for SK-BR-3 parental cells or 1.5×10^4^ cells per well for NCI-N87 parental cells in medium containing 2% FBS. After incubation overnight, recombinant heregulin was added at increasing concentrations with or without a fixed dose of an anticancer drug (lapatinib, trastuzumab, T-DM1 or paclitaxel). The fixed doses were determined to be the lowest dose for maximal growth inhibition as estimated by the results of a growth inhibition assay (Figure 3). After incubation for 72 hours, cell viability was measured using Cell Counting Kit-8 solution (Dojindo, Kumamoto, Japan) according to the manufacturer's instructions.

### In vitro growth inhibition assay in Mock and HRG cells

Cells were seeded into 96-well flat-bottom plates at 4.0×10^3^ cells per well for SK-BR-3 Mock cells and HRG cells, 1.5×10^4^ cells per well for NCI-N87 Mock cells or 1.0×10^4^ cells per well for NCI-N87 HRG cells in RPMI medium containing 2% FBS. After incubation for 24 hours, lapatinib, trastuzumab, T-DM1 or paclitaxel was added at increasing concentrations. After incubation for 72 hours, cell viability was measured using Cell Counting Kit-8 solution according to the manufacturer's instructions.

### Annexin V binding assay to assess apoptosis

Cells were seeded in 10 cm dishes at 1.4×10^6^ cells per dish for 24 hours in RPMI medium supplemented with 2% FBS, and anticancer drugs were added to the conditioned media. After incubation for 48 hours, the floating and attached cells were collected and suspended in 100 μl of Annexin V-FLUOS labeling solution (Roche, Basel, Switzerland). Then, cellular fluorescence was analyzed using a BD FACSCanto II system and BD FACSDiva software (Becton Dickinson, USA).

### Immunoblotting analysis to evaluate intracellular signaling and cell apoptosis

Cells were seeded in 60 mm dishes at a density of 1.5×10^6^ cells per dish for 24 hours in RPMI medium supplemented with 2% FBS. Then, anticancer drugs were added to the conditioned media. After incubation for 24 hours (for analysis of intracellular signaling) or 48 hours (for analysis of cell apoptosis), immunoblotting analysis was performed utilizing antibodies against EGFR, phosphorylated EGFR, phosphorylated HER2, phosphorylated HER 3 (Tyr1289), Akt, phosphorylated Akt (Ser473), ERK, phosphorylated ERK, heregulin, BIM, PARP (all from Cell Signaling Technology, MA, USA), HER2 (Millipore, MA, USA), HER3 (Santa Cruz Biotechnology, CA, USA), survivin (Novus Biologicals, CO, USA) and β-actin (Sigma-Aldrich, MO, USA).

### Tumor growth inhibition assay in vivo

All animal experiments were performed in accordance with the Recommendations for Handling of Laboratory Animals for Biomedical Research compiled by the Committee on Safety and Ethical Handling Regulations or Laboratory Animal Experiments, Kindai University. The study was also reviewed and approved by the Animal Ethics Committee of Kindai University. NCI-N87 Mock and HRG cells were subcutaneously (5 × 10^6^ per mouse) injected into the right flank of female BALB/cAJcl-nu/nu mouse (CLEA Japan). Treatments were initiated when tumors of 5 mice achieved an average volume of 200 to 400 mm^3^. Control mice were administered 100 μl of 0.5% of hydroxypropyl methylcellulose once daily by oral gavage and intraperitoneally injected with 100 μl of PBS twice per week. Lapatinib (100 mg/kg) was dissolved in 100 μl of 0.5% hydroxypropyl methylcellulose and administered once daily by oral gavage. Trastuzumab (0.3 mg/kg body weight in 100 μl PBS) was intraperitoneally injected twice per week. T-DM1 (10 mg/kg body weight in 100 μl PBS) was intraperitoneally injected once on the first day. Treatment groups consisted of 5 mice per group. Tumor volume was determined from caliper measurements of tumor length (L) and width (W) according to the formula LW^2^/2. Both tumor size and body weight were measured twice weekly.

### Quantitative real-time PCR of mRNA expression in tissue samples

Tissue samples were obtained either before or after trastuzumab-based therapy from patients with HER2-positive breast or gastric cancer who were previously treated by the Kindai University Faculty of Medicine after the study was approved by the Institutional Review Board; the patients provided written informed consent. Total RNA was isolated from paraffin-embedded tissues using the RNeasy Mini Kit and the RNeasy FFPE kit (both from Qiagen, CA, USA) according to the manufacturer's instructions. cDNA was prepared using a High Capacity RNA-to-cDNA Kit (Applied Biosystems, CA, USA), and real-time PCR was conducted to assess the expression of heregulin, along with β-actin as an internal standard, using TaqMan (R) Gene Expression Assays (Applied Biosystems, CA, USA). Primers for heregulin (Hs00247620_m1) and β-actin (Hs01060665_g1) were purchased from Thermo Fisher Scientific (MA, USA). Fluorescence was detected using a StepOnePlus Real-Time PCR System (Applied Biosystems, CA, USA). The final results were calculated using the *ΔΔ*Ct method, normalized to the levels of β-actin as an internal control, and standardized to the median value for each sample. PCR efficiency was evaluated via serial dilution of SK-BR-3 HRG cell cDNA, and the efficiencies were 93% and 91% for β-actin and heregulin, respectively.

## CONCLUSION

mRNA up-regulation of heregulin was observed in clinical breast cancer specimens during trastuzumab-based treatment, but heregulin overexpression had a limited effect on sensitivity to T-DM1 in vitro and in vivo.

## SUPPLEMENTARY MATERIALS FIGURES AND TABLES


