# Supplementary material for: Heregulin-expressing HER2-positive breast and gastric cancer exhibited heterogeneous susceptibility to the anti-HER2 agents lapatinib, trastuzumab and T-DM1
Source: Oncotarget. 2016 Oct 19;7(51):84860–71. doi: 10.18632/oncotarget.12743 (PMC5356704; doi:10.18632/oncotarget.12743)
Supplement: Supplementary file 1 [file oncotarget-07-84860-s001.pdf]

Heregulin-expressing HER2-positive breast and gastric cancer exhibited heterogeneous susceptibility to the anti-HER2 agents lapatinib, trastuzumab and T-DM1

SUPPLEMENTARY FIGURES

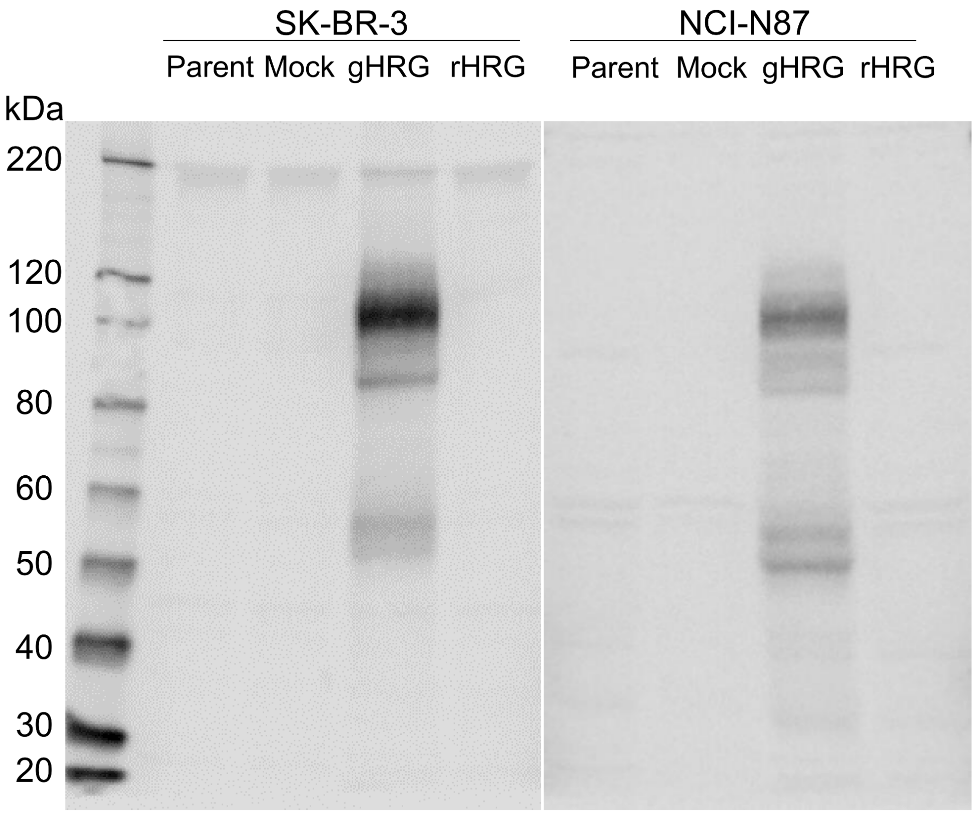

Supplementary Figure S1: Heregulin expression in SK-BR-3 and NCI-N87 parent, Mock, HRG and rHRG cells. SK-BR-3 and NCI-N87 cell lines and their derivatives (SK-BR-3 Mock, SK-BR-3 HRG, NCI-N87 Mock and NCI-N87 HRG) were cultured overnight in medium containing 10% FBS and then incubated for 48 hours in medium containing 2% FBS. Recombinant heregulin was added to parental SK-BR-3 and NCI-N87 cells at 20 ng/ml. After 15 minutes, the cells were lysed and subjected to immunoblotting analysis. Mock=empty vector-transfected cells, gHRG=*heregulin*-transfected cells, rHRG=recombinant heregulin-treated parental cells.

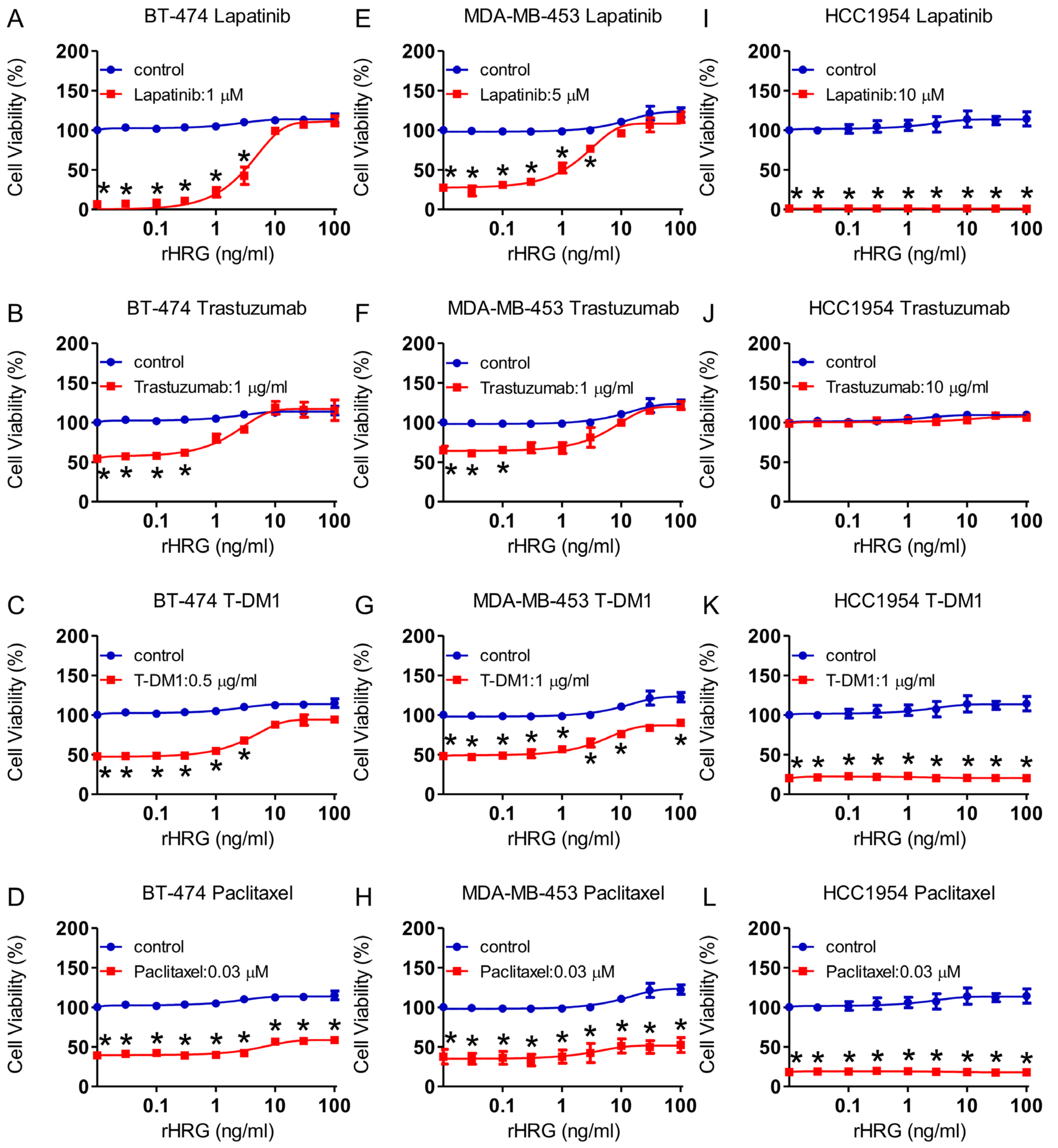
A
BT-474 Lapatinib
control
Lapatinib:1 μM
E
MDA-MB-453 Lapatinib
control
Lapatinib:5 μM
I
HCC1954 Lapatinib
control
Lapatinib:10 μM
B
BT-474 Trastuzumab
control
Trastuzumab:1 μg/ml
F
MDA-MB-453 Trastuzumab
control
Trastuzumab:1 μg/ml
J
HCC1954 Trastuzumab
control
Trastuzumab:10 μg/ml
C
BT-474 T-DM1
control
T-DM1:0.5 μg/ml
G
MDA-MB-453 T-DM1
control
T-DM1:1 μg/ml
K
HCC1954 T-DM1
control
T-DM1:1 μg/ml
D
BT-474 Paclitaxel
control
Paclitaxel:0.03 μM
H
MDA-MB-453 Paclitaxel
control
Paclitaxel:0.03 μM
L
HCC1954 Paclitaxel
control
Paclitaxel:0.03 μM
Cell Viability (%)
rHRG (ng/ml)
0
50
100
150
200
0.1
1
10
100

(*Continued*)

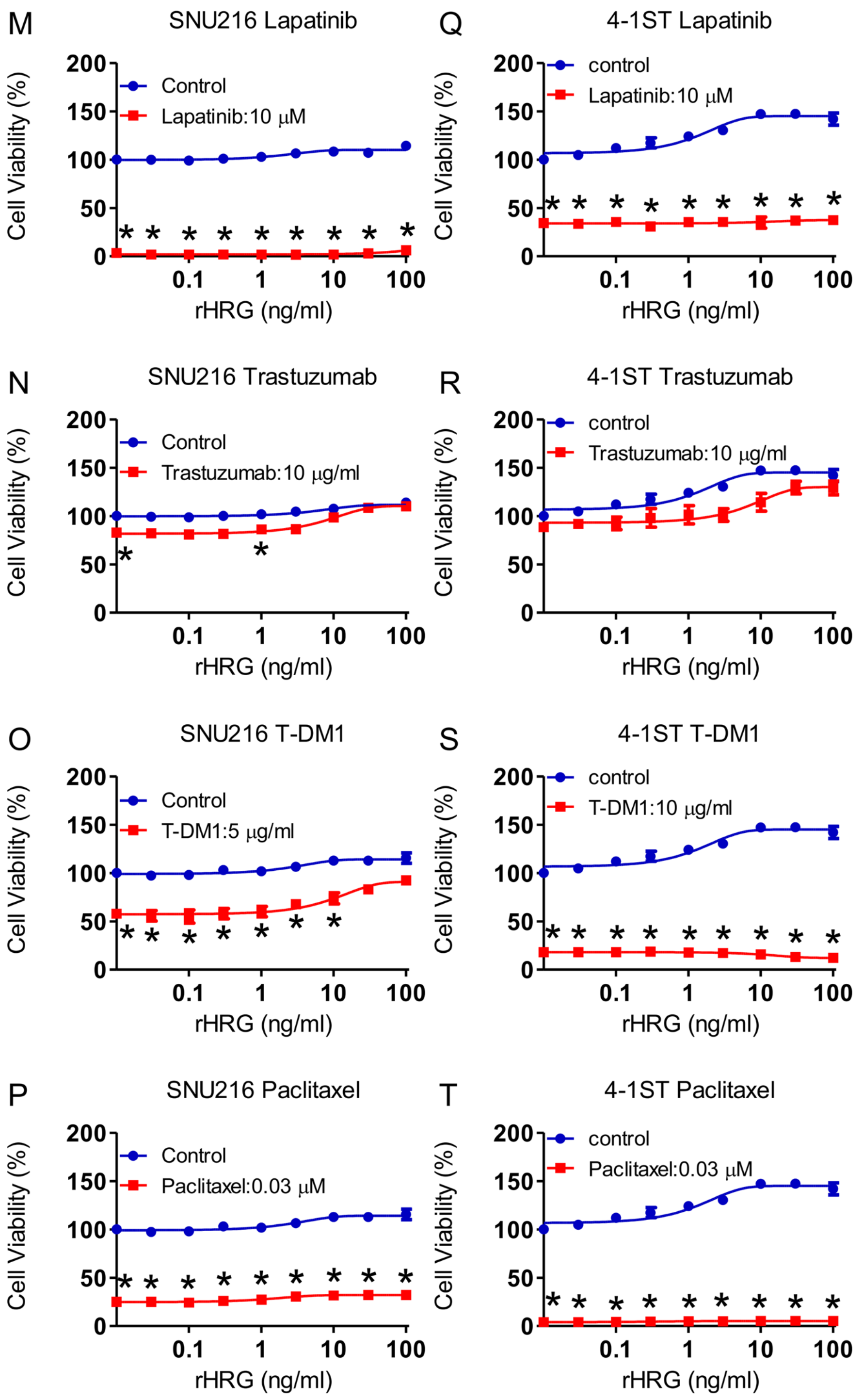

Supplementary Figure S2: Addition of recombinant heregulin and drug resistance in BT-474, MDA-MB-453, HCC1954, SNU-216 and 4-1 ST cell lines. Cells were incubated for 72 hours in the presence of anticancer drugs (red line) or the absence of those drugs (blue line) with increasing doses of recombinant heregulin, and cell viability was measured. The fixed doses of lapatinib (1 μM for BT-474, 5 μM for MDA-MB-453, 10 μM for HCC1954, SNU216 and 4-1ST), trastuzumab (1 μg/ml for BT-474 and MDA-MB-453, 10 μg/ml for HCC1954, SNU216 and 4-1ST), T-DM1 (0.5 μg/ml for BT-474, 1 μg/ml for MDA-MB-453 and HCC1954, 5 μg/ml for SNU216, 10 μg/ml for 4-1ST) and paclitaxel (0.03 μM) were the lowest dose resulting in maximum growth inhibition. The p values were calculated using an unpaired Student's t-test, where * indicates p value < 0.0055 as determined by the Bonferroni correction for multiple comparisons.
